# Integration of PCG spectrogram texture and deep features for the diagnosis of heart failure with preserved ejection fraction using heterogeneous stacking ensemble learning

**DOI:** 10.3389/fphys.2025.1694781

**Published:** 2026-01-15

**Authors:** Yineng Zheng, Jian Qin, Fajin Lv, Xia Li, Xingming Guo

**Affiliations:** 1 Department of Radiology, The First Affiliated Hospital of Chongqing Medical University, Chongqing, China; 2 State Key Laboratory of Ultrasound in Medicine and Engineering, Chongqing Medical University, Chongqing, China; 3 Department of Cardiology, The First Affiliated Hospital of Chongqing Medical University, Chongqing, China; 4 Department of Oncology, The First Affiliated Hospital of Chongqing Medical University, Chongqing, China; 5 Key Laboratory of Biorheology Science and Technology, Ministry of Education, College of Bioengineering, Chongqing University, Chongqing, China

**Keywords:** diagnosis model, ensemble learning, heart sounds, HFpEF, phonocardiogram

## Abstract

This study proposes a novel heterogeneous stacking ensemble learning model for the fusion of phonocardiogram (PCG) spectrogram texture and deep features to detect heart failure with preserved ejection fraction (HFpEF), which plays a critical role in the clinical assessment of chronic heart failure. Firstly, the preprocessed PCG signals were transformed into two-dimensional spectrograms using the Gammatone filter for feature extraction. Four first-order base models were subsequently developed, comprising one texture analysis model and three transfer learning models. The texture analysis model was constructed by extracting texture features and integrating them with a support vector machine, with feature selection performed through recursive feature elimination. The transfer learning models were established on the pre-trained ResNet50, InceptionResNetV2, and DenseNet121, where the conventional softmax classifier was replaced with random forests combined with principal component analysis. Finally, a heterogeneous stacking ensemble learning model was proposed to achieve feature fusion and classification, with a multilayer perceptron (MLP) used as the second-order meta learner by integrating the weighted output probabilities of the four base learners. The proposed model achieved an average AUC of 0.933, an accuracy of 0.902, a sensitivity of 0.958, a specificity of 0.843, a precision of 0.968, and an F1 score of 0.923, demonstrating consistent improvements over the baseline models and commonly used deep learning models for HFpEF detection. This study demonstrates the effectiveness of the proposed ensemble strategy based on PCG analysis and its potential for the computer-aided diagnosis of HFpEF.

## 1 Introduction

Chronic heart failure (CHF) is a serious manifestation of advanced cardiac diseases, characterized by impaired cardiac function and circulation. Different subtypes of chronic heart failure exhibit distinct clinical manifestations and symptoms. Accurate identification of these subtypes allows for precise diagnosis and tailored treatment based on individual characteristics. Although diagnosing HFpEF is straightforward in acutely decompensated patients, it becomes more challenging in stable euvolemic patients (Reddy et al., 2018). Echocardiography and B-type brain natriuretic peptide (BNP) are commonly used for diagnosis, but they may not be sufficient for HFpEF (Borlaug and Paulus, 2011), where ventricular systolic function remains preserved. This creates a diagnostic challenge, especially for patients with BNP values between 100 and 500 pg/mL (Chen et al., 2021). Non-specialists might not have immediate access to invasive cardiac catheterization for confirming elevated LV filling pressures. Conducting specialist diagnostic tests on every patient is neither practical nor feasible, as it would be time-consuming, expensive, and often unnecessary.

The graphical representation of sounds caused by the heart mechanical movement during each cardiac cycle, known as a phonocardiogram (PCG) can offer a noninvasive, risk-free, and cost-effective diagnostic tool for detecting CHF. Specific patterns and characteristics of heart sounds can assist clinicians in identifying subtypes of CHF and assessing its severity (Luo et al., 2023). For example, certain murmurs, abnormal heart rhythms, or extra heart sounds may be associated with specific subtypes of heart failure, such as systolic or diastolic dysfunction. Changes in heart sounds, such as the disappearance or alteration of specific sounds, can indicate improvements or worsening of the underlying condition (Drazner et al., 2003). Analyzing heart sound (HS) features can provide valuable diagnostic clues and offer insights into the pathophysiological mechanisms of heart failure (Cao et al., 2020). However, it remains unclear whether these distinctive features in heart sounds can aid in the identification of HFpEF.

## 2 Related studies

### 2.1 The application of PCG to CHF

In recent years, utilizing PCG as a diagnostic tool for the diagnosis of cardiovascular diseases, particularly CHF, has increased and shown promising results (Gao et al., 2020; Gjoreski et al., 2020). We have investigated a machine learning model that integrates time-domain, time-frequency, and nonlinear features of heart sounds to determine whether the heart is in a state of CHF (Zheng et al., 2015). Thakur et al. (2017) found that PCGs can capture critical acoustic signatures associated with haemodynamic changes, offering valuable information about the pathophysiology of heart failure. Gao et al. (2020) proposed GRU model effectively learns features directly from HS signals without relying on expert knowledge, demonstrating the potential of HS analysis in early HF detection. Gjoreski et al. (2020) proposes a method for detecting CHF based on HS signals, which combines expert feature-driven classical machine learning with end-to-end deep learning models. We extracted multi-scale features from heart sounds and employed machine learning techniques to perform heart failure staging (Zheng et al., 2022). The above research indicates that PCG characteristics for CHF hold promise for early diagnosis, while also enhancing the sensitivity and specificity of diagnostic methods. The significance of diagnosing HFpEF stems from the fact it requires different treatment approaches compared to heart failure with reduced ejection fraction (HFrEF). Therefore, it highlights the necessity of accurately classifying and diagnosing the subtypes of heart failure. However, because of the complexity and heterogeneity of HFpEF, existing studies contain fewer reports on the HS analysis for the diagnosis of HFpEF, compared with those assessing the presence of heart failure, with little research into heart failure subtype recognition.

### 2.2 Transfer learning and ensemble learning methods

The success of PCG classification relies heavily on the feature extraction phase in machine learning. Typically, segmentation points of the PCG are used to extract time-domain features as a standard method (Tang et al., 2010), and furthermore, incorporating frequency-domain or nonlinear features can enhance the classification performance of the model (Ghosh et al., 2022). The advancement of time-frequency decomposition techniques (Zeng et al., 2021), including wavelet transform, wavelet packet transform, and variational mode decomposition, has enhanced the feature extraction of PCG signals, and it is possible to explore detailed information from time and frequency domains at different scales. The feature extraction across multiple domains including the time, frequency, time-frequency or nonlinear domains, coupled with multiscale integration of these features, currently represents the most widely adopted approach (Bao et al., 2023; Khan et al., 2020; Fakhry and Gallardo-Antolín, 2024). The accuracy of classification in these traditional approaches is heavily influenced by both the type and quantity of extracted features, as well as the complexity of the classifier design specific to the pattern recognition challenge.

While end-to-end deep learning has demonstrated outstanding performance across various domains by automatically extracting features from PCG spectrograms and eliminating the manual feature extraction step (Ren et al., 2022), it often requires an extensive volume of training data to mitigate overfitting. However, these models exhibit limited generalization capabilities when trained on small datasets, and access to specialized CHF databases containing a wide variety of heart sounds remains restricted. Recently, transfer learning has emerged as a pivotal solution for small-sample classification, enabling the community to address increasingly complex challenges with continuously improving accuracy. Utilizing transfer learning for feature extraction from PCG spectrograms offers a potential solution to enhance HS classification results in small databases (Khan et al., 2021). The extracted feature vectors are then fed into classifiers such as support vector machine (SVM), random forests (RF), convolutional neural networks (CNNs) and long short-term memory networks (LSTM) (Xiao et al., 2020; Zheng et al., 2023), which also has shown significant improvements in classification.

## 3 Proposed methods

**FIGURE 1:**
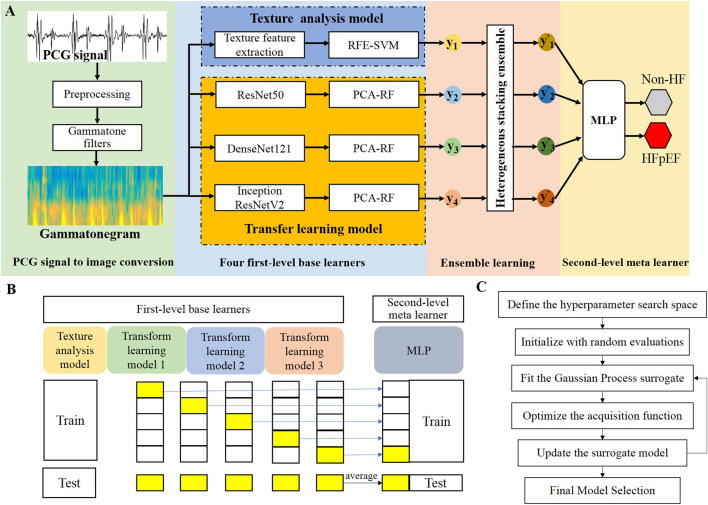
**(A)** Flow chart of this study. $y_{i}\left(i=1,2,3,4\right)$ Denotes the outputs of the base learners, while $y_{i}^{\prime }\left(i=1,2,3,4\right)$ represents the inputs to the meta-learner after heterogeneous stacking ensemble. **(B)** Illustrates the training and test pipeline. **(C)** Presents the Bayesian optimization pipeline.

Given the limited sample size of HFpEF heart sound data, this study employs machine learning for model construction. Leveraging the advantages of transfer learning in small-sample tasks, this study intends to utilize pre-trained CNNs for feature extraction, thereby significantly reduces dependence on handcrafted feature engineering processes. Consequently, one-dimensional PCG signals are converted into two-dimensional spectrograms. Considering that texture analysis provides valuable insights for quantitative image feature characterization, we propose an ensemble learning framework that effectively combines transfer learning and texture analysis models through a stacking heterogeneous ensemble mechanism to discriminate HFpEF. Furthermore, a weighted-based heterogeneous stacking ensemble mechanism is introduced to optimize model integration strategies. The ensemble model integrats the individual classification results of base learners according to a weighted AUC-based integration mechanism, resulting in a fused classification output. The workflow of this study is illustrated in Figure 1A. The proposed model’s effectiveness is further assessed against that of the individual models and baseline models on same database. Thus, the major contributions of this study are as follows:

1. This study proposes a heterogeneous stacking ensemble learning approach based on a weighted AUC-based integration mechanism, and for the first time explores the use of PCG-based ensemble learning for the diagnosis of HFpEF.
2. This study investigates the novel application of texture analysis for feature extraction from PCG spectrograms, effectively integrating both textural and deep features to advance HFpEF diagnosis. This study is also the first to apply texture analysis for heart sound feature extraction.
3. This study validates texture analysis as an effective approach for extracting higher-order statistical features from PCG spectrograms. Furthermore, we demonstrate the efficacy of the proposed ensemble strategy in enhancing comprehensive diagnostic performance through comparative experiments.

## 4 Materials and methods

### 4.1 Datasets

The proposed method was evaluated using two datasets inclluding the public PhysioNet/CinC 2016 open-source database and a private clinical dataset comprising heart failure cases collected at our hospital between March and December 2023. A detailed summary of the heart failure PCG dataset and the PhysioNet/CinC 2016 dataset is presented in Tables 1, 2, respectively. This study received approval from our hospital’s Medical Ethics Committee (approval number 2022-228), and all participants gave written consent. The diagnosis of HFpEF was made by experienced physicians according to expert guidelines. All patients rested for 15–20 min before HS collection. The duration of each collection exceeded 5 min, with a randomly selected 10-s segment of the highest quality signal for each subject.

**TABLE 1:** Description of HFpEF heart sound dataset.

Parameter	Non-heart failure	HFpEF
Subject	136	131
Sample	680	655
Age	56.56 ± 21.43	68.12 ± 17.39
Male/Female	77/59	53/78
BMI	24.51 ± 2.99	24.41 ± 5.74

**TABLE 2:** Description of PhysioNet/CinC 2016 dataset.

Database		Sample size	
		Normal	Abnormal
CinC 2016^a^	Training-a	117	292
	Training-b	386	104
	Training-c	7	24
	Training-d	27	28
	Training-e	1958	183
	Training-f	80	34

^a^ An open source public dataset of heart sounds accessed by https://www.physionet.org/content/-challenge-2016/1.0.0/

### 4.2 Signal preprocessing

First, PCG signals were filtered in the 10–1,000 Hz range with a Butterworth filter to eliminate low-frequency noise and baseline wandering, and then, a fourth-order adaptive FIR notch filter was utilized to eliminate 50 Hz interference. High-frequency noise from artifacts including muscle contractions, electrode movement, friction between the sensor and the skin, respiratory noise or environmental noise were further denoised using the multilevel singular value decomposition and adaptive threshold wavelet transform. Subsequently, each signal was downsampled to 2205 Hz, followed by Z-score normalization.

### 4.3 PCG signal to image conversion

To extract texture and deep features, this study employed gammatone filters to generate 2D time-frequency gammatonegrams from the PCG signals. These filters effectively replicate human auditory perception characteristics, providing a comprehensive and robust time-frequency representation of the signal. In gammatonegrams, lower frequencies have narrower bandwidths, while higher frequencies have broader ones, in contrast to the STFT spectrogram, which employs a constant bandwidth across the entire frequency range (Gupta et al., 2021). The impulse response of a gammatone filter is derived from the product of a Gamma function and a sinusoidal tone using Equation 1:

$$g\left(t,f_{c}\right)=at^{n-1}e^{-2\pi bt}\cos\left(2\pi f_{c}t+\varphi \right)$$ (1)

where $f_{c}$ denotes the filter’s central frequency, t is the time, φ corresponds to the carrier phase and n specifies the filter order. The parameters a and b define the gammatone filter’s amplitude and bandwidth, respectively. By stacking the responses of each frame across overlapping time frames, the gammatonegram spectrogram is ultimately formed using the entire Gammatone filter bank.

### 4.4 Texture feature extraction

Texture analysis was employed to quantify spatial relationships between image voxels in the PCG gammatonegram. A set of classical texture matrices was computed to capture multi-dimensional signal patterns, including the Gray Level Co-occurrence Matrix (GLCM), Gray Level Run Length Matrix (GLRLM), Gray Level Size Zone Matrix (GLSZM), and Gray Level Dependence Matrix (GLDM). All texture features were extracted in accordance with the Image Biomarker Standardization Initiative (IBSI) guidelines, and the mathematical formulations of these matrices are summarized below. Pyradiomics (https://github.com/AIM-Harvard/pyradiomics) was used to extract a total of 70 texture features in this study (Castellano et al., 2004; Van Griethuysen et al., 2017), Specific feature descriptions are provided in Supplementary Material.

The GLCM quantifies the joint occurrence of two gray levels separated by a fixed spatial displacement $\left(\Delta x,\Delta y\right)$ in direction α . Formally, the matrix element $P_{GLCM\left(g_{i},g_{j}\right)}$ is defined as Equation 2:

$$P_{GLCM\left(g_{i},g_{j}\right)}=\#\left\{\left(x,y\right)|I\left(x,y\right)=g_{i}\wedge I\left(x+\Delta x,y+\Delta y\right)=g_{j}\right\}$$ (2)

The matrix is normalized by Equation 3:

$$P_{GLCM\left(g_{i},g_{j}\right)}=\frac{P_{GLCM\left(g_{i},g_{j}\right)}}{\sum_{i,j}P_{GLCM\left(g_{i},g_{j}\right)}}$$ (3)

where $I\left(x,y\right)$ is the gray level of pixel $\left(x,y\right)$ ; g , $g_{i}$ , and $g_{j}$ denote gray-level intensity values. Specifically, g represents a generic gray level in the image, whereas $g_{i}$ and $g_{j}$ correspond to the pair of gray levels whose spatial or structural relationships are quantified by a given texture matrix. These gray-level values are derived from the discretized PCG gammatonegram and are used consistently across all matrix definitions.

GLRLM captures the length of consecutive pixels (runs) with identical gray level g along direction α . A run of length r is defined as a maximal sequence of pixels using Equation 4:

$$I\left(x+k\Delta x,y+k\Delta y\right)=g,\;k=0,1,\ldots ,r-1$$ (4)

where the sequence is terminated when the condition no longer holds. The GLRLM is defined as Equation 5:

$$P_{GLRLM}\left(g,r\right)=\#\left\{\text{runs of length }r\text{ with gray level }g\right\}$$ (5)

GLSZM characterizes the size distribution of homogeneous connected regions (zones) independent of direction. A zone is defined as an 8-connected set of pixels with identical gray level g . The GLSZM entry $P_{GLSZM}\left(g,s\right)$ counts using Equation 6:

$$P_{GLSZM}\left(g,s\right)=\#\left\{\text{zones of size }s\text{ with gray level }g\right\}$$ (6)

where s denotes the number of pixels in the zone.

GLDM quantifies the number of neighboring pixels within direction α whose gray-level difference from the reference pixel does not exceed a tolerance δ . The dependence count is defined as Equation 7:

$$D\left(x,y\right)=\sum_{\left(u,v\right)\in N_{\alpha }}I\left(\left|I\left(x,y\right)-I\left(x+u,y+v\right)\right|\leq \delta \right)$$ (7)

The GLDM element $P_{GLDM}\left(g,d\right)$ is therefore expressed as Equation 8:

$$P_{GLDM}\left(g,d\right)=\#\left\{\left(x,y\right)|I\left(x,y\right)=g\wedge D\left(x,y\right)=d\right\}$$ (8)

where $N_{\alpha }$ is the neighborhood defined along direction α , δ is the gray-level tolerance threshold. The inner summation computes the dependence count, i.e., the number of neighboring pixels whose gray-level difference from the reference pixel is ≤ δ . $P_{GLDM}\left(g,d\right)$ counts the number of pixels with gray level g and dependence value d . “ # ” denotes the cardinality operator, representing the number of pixels satisfying the given condition. The symbol “ ∧ ” denotes the logical AND operator, indicating that both conditions must be simultaneously satisfied. $I\left(\cdot \right)$ denotes the indicator function, which takes the value 1 when the condition is true and 0 otherwise.

### 4.5 Transfer learning

Convolutional neural networks (CNNs), which consist of multiple convolutional layers, excel at capturing spatial or local patterns within images. By utilizing multi-scale filters of varying kernel sizes, they generate diverse high-level representations. To address the challenge of limited dataset size often associated with CNNs, transfer learning can be employed. This approach leverages pre-trained CNNs for deep feature extraction. The PCG gammatonegram spectrogram is subsequently resized for input into the pre-trained CNN model. Previous studies have indicated that some modules such as inception modules, residual networks, and skip connections can enhance model performance. Consequently, this study utilizes three deep transfer learning architectures as feature extractors, namely ResNet50 (He et al., 2016), DenseNet121 (Huang et al., 2017), and InceptionResNetV2 (Szegedy et al., 2017). The Adam optimizer is employed for model fine-tuning, while early stopping is used as a regularization technique to prevent overfitting.

#### 4.5.1 ResNet50

ResNet50 follows the CNN architecture but with a specific variant of the ResNet architecture (He et al., 2016), which used residual learning to address the vanishing gradient. Each layer in a ResNet comprises multiple blocks. The 50-layer ResNet uses a bottleneck architecture in its building blocks, incorporating 1 × 1 convolutions to reduce parameters and matrix multiplications. This approach, which stacks three layers rather than two, accelerates training for each layer.

#### 4.5.2 DenseNet121

DenseNet is a CNN architecture known for its unique cross-layer connectivity pattern. It establishes direct connections between each layer and all subsequent layers (Huang et al., 2017). This connectivity pattern promotes information flow by allowing gradients to be shared across layers during backpropagation, thereby addressing the vanishing gradient issue.

#### 4.5.3 InceptionResNetV2

InceptionResNetV2 architecture is built upon inception blocks, incorporating operations that utilize multiple convolutional filter sizes simultaneously for feature extraction (Szegedy et al., 2017). Bottleneck layers are designed to develop the cross-channel correlations between different paths for reduce computational costs. The architecture combines the advantages of residual networks with the design of inception modules, allowing the model to effectively capture diverse patterns.

### 4.6 Heterogeneous stacking ensemble learning

Traditional ensemble learning methods predominantly depend on averaging and majority voting, neglecting the impact of less effective learners (Polikar, 2012). To address this limitation and achieve more robust results, an ensemble of deep CNNs can be employed, leveraging the combined decisions of multiple models (Islam and Zhang, 2018). Specifically, this study proposes a heterogeneous stacking ensemble learning model.

#### 4.6.1 Heterogeneous base learner construction

To enhance detection accuracy and reduce the errors, it is commonly acknowledged that the diversity of the base learners plays a crucial role in building a high-quality ensemble model (Finn et al., 2017). Therefore, in the selection of base learners, we adopted three various independently pre-trained transfer learning models and one texture analysis model to generate optimal results by integrating multiple classifiers with minimal errors. The transfer learning models leverage pre-trained CNNs to extract deep features, which are then reduced in dimensionality using PCA and fed into a random forest classifier, while the texture analysis model extracts texture features and inputs them into an SVM with recursive feature elimination. Next, we use the outputs of these four distinct classification models as the input for the meta-learner and propose a weighted AUC-based integration mechanism for ensemble learning.

#### 4.6.2 Base learner integration and stacking ensemble classifier

By integrating the outputs of the base learners, a heterogeneous stacking ensemble model was proposed using a weighted AUC-based integration mechanism. The framework incorporates predictions from four distinct base learners, including a texture analysis-based SVM model and three transfer learning-based random forest (RF) models. Ensemble classification enables effective training of the meta learner on a limited number of samples while reducing the risk of overfitting, by leveraging the class posterior probabilities generated by the four base models. Next, we provide a detailed description of the proposed ensemble strategy. Unlike conventional stacking methods that treat all base learners equally, our approach introduces a weighted integration mechanism based on the AUC performance of each base model. Specifically, the class posterior probabilities output by each base learner are first calibrated and then weighted according to their validation AUC scores, allowing models with stronger discriminative power to contribute more significantly to the final decision. These weighted outputs are concatenated and used as the input for the meta learner, which is trained to learn the optimal decision boundaries by capturing high-level interactions among the base learners. This design enhances the robustness and generalizability of the ensemble, particularly in scenarios with limited training data.

Let the posterior probability $P_{\mu }\in \left[0,1\right]$ of a base learner $L_{\mu }^{y}\left(x_{i}\right)$ , where $x_{i}\in R^{N}$ and $y\in \left\{-1,+1\right\}$ indicates the ith input and output patterns. $L_{\mu }^{-1}\left(x_{i}\right)$ and $L_{\mu }^{+1}\left(x_{i}\right)$ respectively represents the forecasted outputs of the μth base learner for class −1 and class +1, where $L_{\mu }^{-1}\left(x_{i}\right)+L_{\mu }^{+1}\left(x_{i}\right)=1$ . Next, a detailed description of the weighted-based heterogeneous stacking ensemble mechanism was presented. The ith element ζ of the input vector for the meta learner can be expressed as Equation 9

$$\zeta_{i}=\omega_{i}P_{i}i=1,2,...,\mu$$ (9)

where μ is the number of base learners, $\omega_{i}$ is the weight of the ith base learner. Since an AUC value evaluates overall classifier performance and reflects generalizability, it is well-suited as a weight for assigning to the corresponding base learners. Let $\hbar_{i}$ denotes the AUC of the ith base learner, $\omega_{i}$ can be calculated by Equation 10

$$\omega_{i}=\hbar_{i}/\sum_{\mu =i}^{\mu }\hbar_{i}$$ (10)

Since this study ultimately includes one texture analysis model and three transfer learning models as base learners, μ is set to 4. The posterior probabilities of each base learner that was incorporated by the corresponding weight have been used as input to the meta learner. A multilayer perceptron composed of three hidden layers, with 256, 128, and 64 neurons in each layer respectively, has been utilized as the meta learner. Finally, through stacking ensemble learning, outputs from the four classifiers are fused to determine the ultimate classification result.

### 4.7 Bayesian optimization

This framework involves numerous weights and hyperparameters, such as batch size and learning rate, which need to be determined and optimized individually. This study applied Bayesian optimization to hyperparameter tuning (Frazier, 2018). Bayesian optimization utilizes prior observations of a loss function l to determine the optimal sampling point. It assumes that the loss function l follows a Gaussian process, which provides an analytically tractable posterior distribution over the loss function l . This setup enables updates to the loss function $l\left(\hat{\eta }\right)$ as new losses are computed for additional hyperparameter sets $\hat{\eta }$ . The algorithm flow of Bayesian optimization can be summarized as follows:

1. Given observed values of $l\left(\eta \right)$ , update the posterior expectation of l using the Gaussian process model.
2. Find new $\hat{\eta }$ that maximizes

$$\text{EI}\left(\hat{\eta }\right)=E\left[\max\left\{0,l\left(\eta \right)-l\left(\hat{\eta }\right)\right\}\right]$$ (11)

3. Compute the loss function for $l\left(\hat{\eta }\right)$ .

All relevant parameters for each ensemble or baseline classification model were optimized, and each weak learner within the ensemble models was independently tuned in each fold. This approach facilitates the comprehensive testing and training of model on the entire dataset, providing a more generalized and more impartial assessment of model performance compared to a simple training and testing set separation.

## 5 Experiments

The public PhysioNet/CinC 2016 database was used to evaluate the model’s baseline performance, while the heart failure PCG dataset was used to assess the model’s ability to distinguish heart failure. Each dataset was split into a training set and an independent testing set with a 7:3 ratio using stratified sampling, ensuring that heart sound segments from the same subject appeared only in either the training or the testing set. Five-fold cross-validation was used on the training set for model development, while model performance was evaluated on the testing set. The data splitting was repeated 10 times with different partitions, and the experiments were repeated 10 times; the average results were taken as the final model performance. In stacking ensemble learning, after splitting the dataset into training and testing sets, the base learners are trained using K-fold cross-validation on the training set. For each fold, base learners are trained on K-1 folds and generate predictions on the held-out fold, creating out-of-fold predictions for the entire training set, and then these out-of-fold predictions serve as input features to train the meta learner, as shown in Figure 1B. After training, base learners are applied to testing set for producing base learner predictions, and finally the meta learner uses these base learner predictions as input to generate the final predictions on the testing set. Bayesian optimization (Figure 1C) was applied to tune the hyperparameters, and the final experimental parameter settings are shown in the Table 3. To assess whether the predictive performance differed significantly between models, pairwise comparisons of AUCs were conducted using the nonparametric DeLong test, which provides a distribution-free estimate of the variance and covariance of correlated ROC curves.

**TABLE 3:** Experimental parameter settings.

Parameter	Value
Optimizer	Adam
Batch size	16
Epoch	120
Learning rate	0.0001

## 6 Results

### 6.1 Texture analysis construction

Table 4 presents the performance of different machine learning models used as base learners for HFpEF diagnosis. It can be seen that k-Nearest Neighbor (KNN) and logistic regression (LR) achieved relatively poor classification performance, while RF and SVM using texture features significantly outperformed KNN and LR in identifying HFpEF. Moreover, SVM outperformed RF in terms of AUC, accuracy, specificity, and F1 score, achieving 0.835, 0.829, 0.931, and 0.839 on the testing set, respectively. Therefore, through comparison, the texture analysis-based SVM-RFE is selected as the first base classifier.

**TABLE 4:** The classification performance of different classifiers using texture features on HF dataset.

Classifier	Dataset	AUC [95% confidence interval]	Accuracy	Sensitivity	Specificity	Precision	F1 score
KNN	Training set	0.864 [0.829–0.9062]	0.821	0.806	0.84	0.806	0.833
	Testing set	0.743 [0.681–0.807]	0.766	0.695	0.882	0.669	0.772
LR	Training set	0.897 [0.852–0.932]	0.855	0.877	0.824	0.877	0.877
	Testing set	0.779 [0.788–0.832]	0.786	0.887	0.651	0.887	0.825
RF	Training set	0.932 [0.889–0.954]	0.864	0.864	0.890	0.846	0.889
	Testing set	0.812 [0.754–0.871]	0.759	0.838	0.733	0.838	0.826
SVM	Training set	0.947 [0.915–0.971]	0.882	0.838	0.945	0.838	0.893
	Testing set	0.835 [0.779–0.8853]	0.829	0.792	0.931	0.792	0.839

### 6.2 Construction and selection of base learners

**FIGURE 2:**
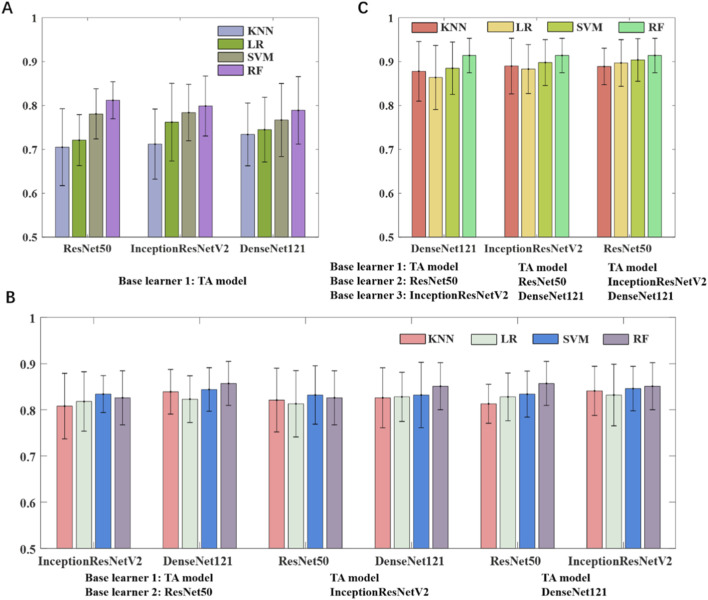
Model performance of ensemble learning models using different base learners. **(A)** Texture analysis model and one transfer learning model. **(B)** Texture analysis model and two transfer learning models. **(C)** Texture analysis model and three transfer learning models.

In this study, we developed two categories of base learners: one based on texture analysis and the other leveraging transfer learning. The machine learning classifiers and the number of base learners used in the ensemble were determined through different combination strategies. We first adopted a stacking strategy in which a two-layer MLP (256 neurons per layer) was used to fuse the outputs of the base classifiers. As shown in Figure 2A, combining two base learners, namely an SVM-based texture model and an RF-based transfer learning model, outperformed either individual model and achieved an AUC of 0.812 on the testing set. Increasing the number of base learners to three (Figure 2B) by integrating the texture model with two RF-based transfer learning models, the AUC of the model improved to 0.857. As shown in Figure 2C, the best performance was obtained when one texture model and three transfer learning models were combined, yielding an AUC of 0.914. Through stacking ensemble learning, the outputs of all four base classifiers were effectively fused to generate the final prediction.

### 6.3 Meta learner construction

**FIGURE 3:**
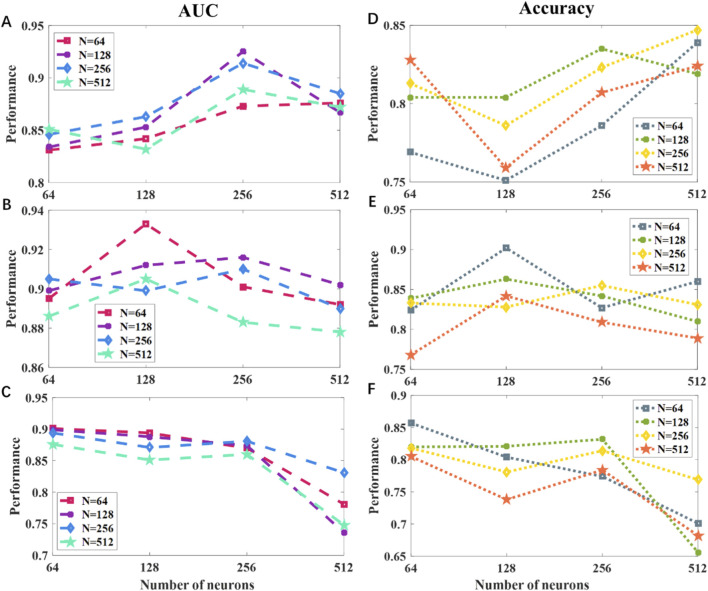
The impact of the number of hidden layers and neurons per layer on the performance of the ensemble model for the meta learner. **(A–C)** The relationships between the model’s AUC and the number of neurons with two, three and four hidden layers. **(D–F)** The relationships between the model’s accuracy and the number of neurons with two, three and four hidden layers.

This study developed four base classifiers, which are an RFE-SVM based on texture analysis of PCG spectrogram, and three PCA-RFs based on deep features from ResNet50, InceptionResNetV2 and DenseNet121, respectively. After determining the number and type of base learners, we compared the impact of using LR and MLP as meta learners on the performance of the ensemble model. The ensemble strategy was consistent with the previous section. As shown in Table 5, the classification performance of the MLP as a meta learner was superior to that of LR. Furthermore, we evaluated how different hidden-layer configurations influence the performance of the MLP meta-learner. As shown in Figure 3, a two-layer MLP with 256 and 128 neurons achieved an AUC of 0.914 and an accuracy of 0.833. Adding a third hidden layer further improved overall performance, with the best results obtained when the additional layer contained 64 neurons (AUC = 0.933, accuracy = 0.902). However, deeper architectures led to performance degradation. Accordingly, the final meta-learner was configured as a three-layer MLP with 256, 128, and 64 neurons.

**TABLE 5:** The classification performance of different meta learners.

Meta learner	Dataset	AUC [95% confidence interval]	Accuracy	Sensitivity	Specificity	Precision	F1 score
LR	Training set	0.935 [0.901–0.963]	0.878	0.831	0.945	0.831	0.889
	Testing set	0.883 [0.847–0.948]	0.828	0.854	0.791	0.854	0.854
MPL	Training set	0.969 [0.941–0.982]	0.918	0.938	0.879	0.891	0.925
	Testing set	0.915 [0.877–0.953]	0.843	0.901	0.872	0.827	0.913

### 6.4 Comparison of ensemble strategies

**FIGURE 4:**
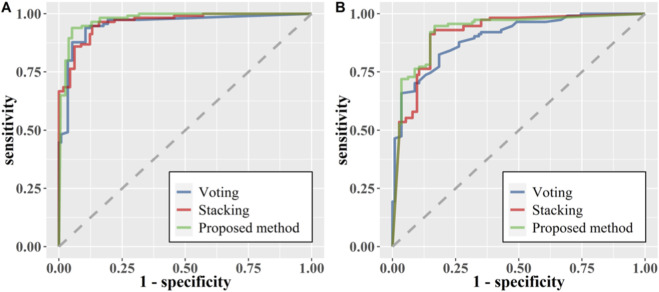
ROC curves of ensemble models based on different fusion strategies. **(A)** Training set. **(B)** Testing set.

To assess the effectiveness of our proposed ensemble strategy, we compared it with two alternative integration methods: voting and stacking. Table 6 presents the performance of classification models employing various integration strategies on the training and testing sets. The result reveal that the performance of ensemble learning models based on stacking and voting are relatively similar in terms of specificity, but the stacking-based ensemble learning model is superior, achieving an AUC of 0.914, an accuracy of 0.872, a sensitivity of 0.915, a specificity of 0.843, a precision of 0.859, and an F1 score of 0.892. The voting-based ensemble learning model achieves the worst performance on those indicators. Furthermore, the heterogeneous stacking-based ensemble learning model proposed in this study achieves the highest classification performance across all metrics, with an AUC, accuracy, sensitivity, specificity, precision, and F1 score of 0.933, 0.902, 0.958, 0.843, 0.968, and 0.923, respectively. The ROC curves of the three ensemble models are shown in the Figure 4.

**TABLE 6:** Performance comparison of ensemble models based on different integration strategies on training and testing sets.

Ensemble strategy	Dataset	AUC [95% confidence interval]	Accuracy	Sensitivity	Specificity	Precision	F1 score
Voting	Training set	0.954 [0.925–0.981]	0.897	0.921	0.890	0.899	0.915
	Testing set	0.897 [0.852–0.934]	0.820	0.824	0.815	0.812	0.827
Stacking	Training set	0.962 [0.940–0.979]	0.911	0.947	0.868	0.886	0.923
	Testing set	0.914 [0.872–0.950]	0.872	0.915	0.843	0.859	0.892
Proposed method	Training set	0.975 [0.953–0.992]	0.941	0.942	0.939	0.945	0.951
	Testing set	0.933 [0.899–0.959]	0.902	0.958	0.843	0.968	0.923

### 6.5 Ablation study

To evaluate the contribution of each component in the proposed heterogeneous ensemble architecture, we conducted a series of ablation experiments by removing (i) the meta-learner, and (ii) individual CNN feature extractors (ResNet50, DenseNet, and InceptionResNetV2). The results are summarized in Table 7. Removing the MLP meta-learner produced the largest performance drop, demonstrating its essential role in integrating heterogeneous radiomic and deep feature representations. Removing DenseNet led to the greatest degradation among CNN branches, indicating its strong complementary contribution to the spectral encoding of HFpEF-related PCG patterns. Models trained with CNN-only features showed substantially lower performance, confirming the necessity of feature fusion. The full model achieved the best overall performance. These results collectively validate the robustness and design rationale of the proposed ensemble system.

**TABLE 7:** Results of the ablation studies.

Model	AUC	Accuracy	Sensitivity	Specificity	Precision	F1 score
Without MLP using average voting	0.882^a^	0.807	0.866	0.842	0.792	0.846
Without ResNet50	0.861^a^	0.791	0.843	0.812	0.773	0.82
Without DenseNet	0.879^a^	0.803	0.862	0.837	0.788	0.842
Without InceptionResNetV2	0.887^a^	0.812	0.87	0.846	0.799	0.852
CNN Features Only	0.892	0.815	0.874	0.851	0.804	0.856
Full Model	0.933	0.902	0.958	0.843	0.968	0.923

^a^ Indicates a statistically significant difference based on the DeLong test when compared with the AUC of the proposed method using heart failure database.

### 6.6 Model comparison

To benchmark our approach against widely used CNN architectures, we evaluated six representative models, such as ResNet50, DenseNet, InceptionResNetV2, MobileNet, ShuffleNet V2, and GhostNe, on both the public PhysioNet/CinC 2016 database and our private clinical heart failure dataset. As summarized in Table 8, the proposed method consistently outperformed all baseline CNN models across both datasets, demonstrating superior discriminative capability.

**TABLE 8:** Performance comparison of the proposed heterogeneous stacking ensemble learning model with deep learning approaches on the testing set.

Database	Model	AUC	Accuracy	Sensitivity	Specificity	Precision	F1 score
PhysioNet/CinC 2016 database	ResNet50	0.873^#^	0.812	0.804	0.825	0.791	0.797
	DenseNet	0.881	0.819	0.811	0.833	0.799	0.805
	InceptionResNetV2	0.889	0.824	0.816	0.839	0.806	0.811
	MobileNet	0.862^#^	0.803	0.792	0.818	0.775	0.783
	ShuffleNet V2	0.857^#^	0.798	0.785	0.812	0.768	0.776
	GhostNet	0.868^#^	0.807	0.798	0.821	0.782	0.789
	Proposed method	0.915	0.843	0.901	0.872	0.827	0.913
Heart failure database	ResNet50	0.906	0.872	0.873	0.870	0.876	0.882
	DenseNet	0.915	0.887	0.952	0.917	0.928	0.931
	InceptionResNetV2	0.894^*^	0.843	0.879	0.800	0.818	0.855
	MobileNet	0.879^*^	0.837	0.880	0.808	0.824	0.857
	ShuffleNet V2	0.881^*^	0.824	0.848	0.817	0.826	0.842
	GhostNet	0.874^*^	0.797	0.801	0.792	0.789	0.804
	Proposed method	0.933	0.902	0.958	0.843	0.968	0.923

# and * indicates a statistically significant difference based on the DeLong test when compared with the AUC, of the proposed method using PhysioNet/CinC 2016 database and heart failure database, respectively.

## 7 Discussion

This study proposed a heterogeneous stacking ensemble learning model that integrates texture analysis and transfer learning for the diagnosis of HFpEF. To the best of our knowledge, this is the first study to utilize texture analysis for feature extraction from heart sounds and to explore the diagnosis of HFpEF using an ensemble learning framework based on PCG signals. Texture features capture detailed relational patterns within the PCG spectrogram, whereas deep representations extracted via transfer learning provide more abstract and complementary information. Comprising an RFE-SVM texture model and three PCA-RF transfer learning models, effectively combines the strengths of heterogeneous base learners to enhance diagnostic performance.

The treatment plans for HFpEF and HFrEF are completely different (Redfield et al., 2023). Thus, the findings of this study hold substantial value in aiding the auxiliary diagnosis of HFpEF and supporting clinicians in patient management. PCG are a direct reflection of the mechanical activity of the heart. HFpEF primarily results from reduced myocardial compliance and impaired diastolic function, both of which are indicative of declining cardiac mechanics. Consequently, PCG deep feature representation and feature fusion could potentially offer enhanced diagnostic capabilities for HFpEF. The advantage of texture analysis in this context is highlighted by its ability to extract detailed patterns and features from PCG gammatonegrams, which can enhance the accuracy and robustness of diagnostic models. GLCM contrast/entropy reflects increased cycle-to-cycle variability in high-frequency components, which is consistent with impaired relaxation and altered filling dynamics in HFpEF. GLRLM short-run features capture transient acoustic irregularities associated with stiff ventricular walls and abnormal diastolic sound patterns. GLDM dependence entropy characterizes localized complexity in the gammatonegram, which increases in HFpEF due to disrupted ventricular suction and elevated filling pressures.

PCG classification is a key component of intelligent auscultation and supports the diagnosis and risk assessment of cardiovascular diseases. Table 9 summarizes representative studies that employ hybrid ensemble frameworks and deep learning models for heart sound analysis. Most approaches focus on improving classification performance through model ensembling (She and Cheng, 2022). Noman et al. (2019) proposed a deep CNN framework combining 1D and 2D CNNs into a time–frequency ensemble. Chen et al. (2022) evaluated multi-resolution models and incorporated ensemble learning to boost accuracy. Potes et al. (2016) extracted statistical and wavelet-based features for an AdaBoost classifier, trained CNNs on frequency-band–decomposed PCG cycles, and fused both outputs. Baydoun et al. (2020) used statistical and wavelet features with bagging and boosting to enhance ensemble performance. Singh et al. (2024) introduced a transfer learning–based ensemble using spectrogram images and STFT features to improve robustness and accuracy. To our knowledge, this is the first study to integrate handcrafted texture features with transfer learning–derived deep features from PCG gammatonegrams within an ensemble learning framework for HFpEF diagnosis. The results demonstrate that combining texture and deep representations yields superior performance and effectively improves HFpEF discrimination.

**TABLE 9:** The summary of ensemble learning for heart sound classification during recent years.

Author (year)	Methods	Model	Application	Findings
She and Cheng (2022)	Mel- frequency cepstral coeffcients	Hybrid ensemble identifcation network using RF, CART, Bagging, XGBoost, SVC and ExtraTrees	Normal/abnormal classification	Accuracy: 96.37% on MIT-BIH database Accuracy: 99.56% on HSCT11 database
Noman et al. (2019)	Mel-frequency cepstral coefficients	time-frequency CNN ensemble combining the 1D-CNN and 2D-CNN	Normal/abnormal classification	Accuracy: 89.22% Sensitivity: 89.94%
Chen et al. (2022)	Time domain, frequency domain, and statistical characteristics	SVM, Random Forest, and KNN are the primary classifiers as the first layer, and the secondary is a logistic regression model	Normal/abnormal classification	Accuracy: 97.28% Sensitivity: 98.45% Specificity: 96.12% Overall score: 97.29%
Potes et al. (2016)	Time-frequency features such as S1, systole, S2, diastole, discrete-time Fourier transform, power across nine frequency bands, etc.	Ensemble of classifiers combining the outputs of AdaBoost and the CNN	Normal/abnormal classification	Sensitivity: 94.24% Specificity: 77.81% Overall score: 86.02%
Baydoun et al. (2020)	Statistical components, such as the mean and standard deviation, wavelet-based features	bagging and boosting ensemble classifier	Normal/abnormal classification	Accuracy: 86.6% Sensitivity: 90.1% Specificity: 83.2%
Singh et al. (2024)	STFT based spectrogram features	The ensemble methodology comprising AlexNet, SqueezeNet, and VGG19 models	Normal/abnormal classification	Accuracy: 99.20% Sensitivity: 99.47%

In the field of medical artificial intelligence research, more and more studies are focusing on the computer-aided diagnosis of HFpEF using advanced supervised algorithms. Table 10 provides an overview of several related studies previously published about the computer-aided diagnosis of HFpEF using medical data and information. Because these studies used different datasets, patient populations, acquisition conditions, and outcome definitions, the results are not directly comparable to the present work. This table is intended only to provide general context regarding prior methods rather than a head-to-head performance comparison. The clinical decision system demonstrated high diagnostic accuracy for heart failure, achieving a 98.3% concordance rate with specialists in a retrospective test dataset and 98% in a prospective pilot study. These findings suggest that AI-CDSS could significantly aid in heart failure diagnosis, especially in the absence of heart failure specialists (Choi et al., 2020). Kwon et al. (2021) developed and validated a deep learning model by using electrocardiography (ECG), showing high performance in detecting HFpEF. The model’s performance indicates that conventional and diverse life-type ECG devices can effectively screen for HFpEF and help prevent disease progression. Ward et al. (2022) developed a machine learning-based ensemble model that demonstrated strong diagnostic performance for HFpEF using demographic and plasma biomarker data, offering a promising noninvasive diagnostic tool and identifying key biomarkers for further study. Akerman et al. (2023) used 3D CNNs to analyze single apical 4-chamber transthoracic echocardiogram video clips for detecting HFpEF. The model showed excellent performance in distinguishing HFpEF from non-HFpEF, outperforming traditional clinical scores and identifying patients who have increased mortality rates. Wu et al. (2024) investigated the use of natural language processing to enhance the detection and diagnosis of HFpEF through electronic health records (EHR).

**TABLE 10:** The summary of artificial intelligence in the diagnosis of HFpEF during recent years.

Author (year)	Methods	Model	Application	Findings
Choi et al. (2020)	Demographics, symptoms, signs, medical history, laboratory examination, electrocardiography, and echocardiography	Expert-driven approach ML-driven approach AI-CDSS	Diagnosis of HFpEF	Accuracy: 82%, 78.9% and 99.5% for three models, respectively
Kwon et al. (2021)	12-lead ECG	Deep learning model	Detection of HFpEF	AUC: 0.866 [95% CI, 0.850–0.883] on internal validation AUC: 0.869 [95% CI, 0.860–0.877] on external validation
Ward et al. (2022)	Demographic information, plasma biomarkers and echocardiogram data	Machine learning-based ensemble classification algorithm	Diagnosis of HFpEF	AUC: 0.90 using multiple data AUC: 0.88 using only demographic and plasma panel data
Akerman et al. (2023)	Single apical 4-chamber transthoracic echocardiogram	3-D convolutional neural network	Detection of HFpEF	Sensitivity: 87.8% [95% CI, 84.5%–90.9%] and Specificity: 81.9% [95% CI, 78.2%–85.6%] on independent testing set
Wu et al. (2024)	Demographic, clinical, echocardiographic and outcome data	Natural language processing	Diagnosis of HFpEF	Accuracy: 75.4% met ESC criteria diagnosis of HFpEF
Ours	PCG texture analysis and deep learning features	Heterogeneous stacking ensemble learning	Diagnosis of HFpEF	AUC: 0.933 [95% CI, 0.899–0.959] Accuracy: 0.902 Sensitivity: 0.958 Specificity: 0.843

ECG, electrocardiography, CI: confidence interval, AI-CDSS: Artificial Intelligence-Clinical Decision Support System.

Most existing studies rely on multimodal fusion of large-scale health data, typically integrating clinical information, laboratory indicators, and features from electrocardiograms or echocardiograms. In contrast, our study takes an alternative approach by focusing exclusively on PCG signals. By extracting physiological and pathological information directly related to the mechanical activity of the heart, the proposed method aims to facilitate non-invasive detection of HFpEF. Despite a relatively small sample size, comparable classification performance was achieved through high-dimensional texture and deep feature extraction from PCG spectrograms combined with a heterogeneous ensemble learning model. These findings highlight the diagnostic potential of heart sounds for HFpEF.

This study also has several limitations. First, the HFpEF database used in this study is limited by a single-center cross-sectional design with a small sample size. Second, the focus of the study is primarily on diagnostic accuracy, with insufficient exploration of the model’s long-term clinical implications, such as its impact on patient outcomes. Future research should include longitudinal studies to evaluate how this diagnostic tool affects clinical outcomes. Third, the exclusion of patients with atrial fibrillation restricts the applicability of our findings to the broader HFpEF population. To enhance the model’s generalizability, larger datasets and external validation from multiple institutions are needed.

## 8 Conclusion

This study explored the application and utility of PCG-based ensemble learning in identifying HFpEF. Firstly, the Gammatone filter was applied to PCG signals for two-dimensional spectrogram transform, followed by feature extraction using texture analysis and transform learning by the pre-trained ResNet50, InceptionResNetV2, and DenseNet121. Texture features were selected via RFE and used to construct an SVM classifier, while deep features were reduced by PCA and modeled using RF classifiers. An MLP meta-learner was then employed to integrate all base classifiers within a heterogeneous stacking framework. The results demonstrate that the proposed ensemble strategy can effectively detect HFpEF from heart sounds, highlighting its potential value for clinical screening and decision support.

## Data Availability

The raw data supporting the conclusions of this article will be made available by the authors, without undue reservation.
