# Supplementary material for: Integration of PCG spectrogram texture and deep features for the diagnosis of heart failure with preserved ejection fraction using heterogeneous stacking ensemble learning
Source: Front Physiol. 2026 Jan 15;16:1694781. doi: 10.3389/fphys.2025.1694781 (PMC12852002; doi:10.3389/fphys.2025.1694781)
Supplement: Supplementary file 1 [file Table1.docx]

Table S1 The description of texture features

| Type | N | Features |
| --- | --- | --- |
| Gray level co-occurrence matrix (GLCM) features | 24 | Autocorrelation, Joint Average, Cluster Prominence, Cluster Shade, Cluster Tendency, Contrast, Correlation, Difference Average, Difference Entropy, Difference Variance, Joint Energy, Joint Entropy, Informational Measure of Correlation1, Informational Measure of Correlation2, Inverse Difference Moment, Maximal Correlation Coefficient, Inverse Difference Moment Normalized, Inverse Difference, Inverse Difference Normalized, Inverse Variance, Maximum Probability, Sum Average, Sum Entropy, Sum of Squares |
| Gray-level size zone matrix (GLSZM) features | 16 | Small Area Emphasis, Large Area Emphasis, Gray Level Non-Uniformity, Gray Level Non-Uniformity Normalized, Size-Zone Non-Uniformity, Size-Zone Non-Uniformity Normalized, Zone Percentage, Gray Level Variance, Zone Variance, Zone Entropy, Low Gray Level Zone Emphasis, High Gray Level Zone Emphasis, Small Area Low Gray Level Emphasis, Small Area High Gray Level Emphasis, Large Area Low Gray Level Emphasis, Large Area High Gray Level Emphasis |
| Gray-level run length matrix (CLRLM) features | 16 | Short Run Emphasis, Long Run Emphasis, Gray Level Non-Uniformity, Gray Level Non-Uniformity Normalized, Run Length Non-Uniformity, Run Length Non-Uniformity Normalized, Run Percentage, Gray Level Variance, Run Variance, Run Entropy, Low Gray Level Run Emphasis, High Gray Level Run Emphasis, Short Run Low Gray Level Emphasis, Short Run High Gray Level Emphasis, Long Run Low Gray Level Emphasis, Long Run High Gray Level Emphasis |
| Gray-level dependence matrix (GLDM) features | 14 | Small Dependence Emphasis, Large Dependence Emphasis, Gray Level Non-Uniformity, Gray Level Non-Uniformity Normalized, Dependence Non-Uniformity, Dependence Non-Uniformity Normalized, Gray Level Variance, Dependence Variance, Dependence Entropy, Low Gray Level Emphasis, High Gray Level Emphasis, Small Dependence Low Gray Level Emphasis, Small Dependence High Gray Level Emphasis, Large Dependence Low Gray Level Emphasis, Large Dependence High Gray Level Emphasis |
